# DAILY WORRY, RUMINATION, AND SLEEP IN LATE LIFE

**DOI:** 10.1093/geroni/igad104.1926

**Published:** 2023-12-21

**Authors:** Zexi Zhou, Kira Birditt, Kate Leger, Karen Fingerman

**Affiliations:** The University of Texas at Austin, Austin, Texas, United States; Institute for Social Research, University of Michigan, Ann Arbor, Michigan, United States; University of Kentucky, Lexington, Kentucky, United States; The University of Texas at Austin, Austin, Texas, United States

## Abstract

Perseverative thinking, including worry and rumination, is a common response induced by stress, and can be detrimental to well-being and health especially for older adults. Sleep may represent an important mechanism by which preservative thoughts affect health. Yet, limited research has investigated links between worry and rumination and their implications for sleep in late life. This study examines the associations between older adults’ everyday worry, rumination, and sleep. We leveraged the daily diary data over 5 to 6 days from the Daily Experiences and Well-being Study (N = 271, Mage = 73.62). Every evening, older adults reported worry and rumination they experienced that day. Every morning, they indicated how worried they were about something that might happen that day, and sleep the prior night (e.g., duration, quality, disturbances). Multilevel models showed that greater evening rumination was associated with fewer sleep disturbances. More hours of sleep, higher sleep quality, and fewer sleep disturbances were associated with less worry the next morning. Greater evening worry and rumination were both associated with greater next morning’s worry, but more hours of sleep buffered the lingering of worry over the night. These results suggest that worry and rumination may tend to persist in older adults’ daily life, and highlight the protective role that better sleep may play in reducing older adults’ everyday perseverative thinking.

